# TALEN and CRISPR/Cas Genome Editing Systems: Tools of Discovery

**Published:** 2014

**Authors:** A. A. Nemudryi, K. R. Valetdinova, S. P. Medvedev, S. M. Zakian

**Affiliations:** Institute of Cytology and Genetics, Siberian Branch of the Russian Academy of Sciences, Lavrentyev Prosp., 10, Novosibirsk, Russia, 630090; Institute of Chemical Biology and Fundamental Medicine, Siberian Branch of the Russian Academy of Sciences, Lavrentyev Prosp., 8, Novosibirsk, Russia, 630090; Meshalkin Novosibirsk State Research Institute of Circulation Pathology, Ministry of Health of the Russian Federation, Rechkunovskaya Str., 15, Novosibirsk, Russia, 630055; Novosibirsk State University, Pirogova Str., 2, Novosibirsk, Russia, 630090

**Keywords:** TALEN, CRISPR/Cas9, genome editing

## Abstract

Precise studies of plant, animal and human genomes enable remarkable opportunities of obtained data application in biotechnology and medicine. However, knowing nucleotide sequences isn’t enough for understanding of particular genomic elements functional relationship and their role in phenotype formation and disease pathogenesis. In post-genomic era methods allowing genomic DNA sequences manipulation, visualization and regulation of gene expression are rapidly evolving. Though, there are few methods, that meet high standards of efficiency, safety and accessibility for a wide range of researchers. In 2011 and 2013 novel methods of genome editing appeared – this are TALEN (Transcription Activator-Like Effector Nucleases) and CRISPR (Clustered Regulatory Interspaced Short Palindromic Repeats)/Cas9 systems. Although TALEN and CRISPR/Cas9 appeared recently, these systems have proved to be effective and reliable tools for genome engineering. Here we generally review application of these systems for genome editing in conventional model objects of current biology, functional genome screening, cell-based human hereditary disease modeling, epigenome studies and visualization of cellular processes. Additionally, we review general strategies for designing TALEN and CRISPR/Cas9 and analyzing their activity. We also discuss some obstacles researcher can face using these genome editing tools.

## INTRODUCTION

Genetic engineering emerged in the laboratory of Paul Berg in 1972 in the form of a recombinant DNA technology, when scientists combined the *E. coli *genome with the genes of a bacteriophage and the SV40 virus. Since then, this science has achieved tremendous success; the molecular genetic mechanisms and phenomena that can now be reproduced *in vitro *have been discovered and studied in detail. Studies in the field of molecular genetics and biochemistry of bacteria and viruses have allowed the development of methods to manipulate DNA, generate various vector systems and methods for their delivery to the cell. All of this has enabled not only transgenic microorganisms production, but also genetically modified plants and animals. The application area of genetic engineering has experienced rapid development, which provided the impetus for progress in selection and biotechnology. However, the conventional genetic engineering strategy has several drawbacks and limitations, one of which is the complexity of manipulations with large animal and human genomes.

From 1990 to 2003, the nucleotide sequence of human nuclear DNA was determined and about 20.5 thousand genes were identified within the “Human Genome” International Project. Similar projects are also currently under implementation; the genome nucleotide sequences of the main model biological objects (*E. coli*, nematode, drosophila, mouse, and others) have been deciphered. However, these projects provide data on the DNA nucleotide sequence only, but they yield no information about function of individual genome elements, or how they interrelate in an entire system. Understanding the functional relationships in the human genome will make it possible not only to identify the cause-and-effect relations in the pathology of hereditary as well as multifactorial diseases, but also to find targets for their treatment.

In 2003, the U.S. National Human Genome Research Institute launched a new international project, ENCODE (Encyclopedia Of DNA Elements), which aim was to join the efforts of scientists and obtain a complete list of the functional elements of the human genome, including the elements that act at the protein and RNA level , as well as the regulatory elements that control the fundamental genetic processes (transcription, translation, and replication). To establish these functional relationships, two strategies are used: switching off a gene (knockout or knockdown) and enhancing the gene activity or its ectopic expression. The traditional methods – transgenesis using homologous recombination in mice [1] and also the use of various viral, including lentiviral vectors– are not only expensive but also quite labor-intensive; they do not allow one to introduce precise changes into a strictly defined genome locus.

Currently, researchers have several tools that allow them to solve the problems of precise plant’s, animal’s, and human’s genome editing .

As early as 1996, a zinc finger protein domain coupled with the FokI endonuclease domain was demonstrated for the first time to act as a site-specific nuclease cutting DNA at strictly defined sites *in vitro* [2]. This chimeric protein has a modular structure, because each zinc finger domain recognizes one nucleotide triplet (zinc finger nuclease, ZFN). This method became the basis for editing cultured cells, including pluripotent stem cells, plant and animal models [3-8]. However, the ZFN-based technology has a number of disadvantages, including the complexity and high cost of protein domains construction for each particular genome locus and the probability of inaccurate cleavage of target DNA due to single nucleotide substitutions or inappropriate interaction between domains. Therefore, an active search for new methods for genome editing was continued. In recent years, this search has led to the development of new tools for genome editing: TALENs (transcription activator-like effector nucleases) and CR ISPR/Cas (clustered regulatory interspaced short palindromic repeats). These systems are characterized by a relative construction simplicity and a high functional efficiency in human, animal, and plant cells. These systems, which are extensively used for various genome manipulations, allow one to solve complex problems, including the mutant and transgenic plants and animals generation, development and investigation of disease models based on cultured human pluripotent cells. Furthermore, chimeric proteins based on the TALE and inactivated Cas9 nuclease DNA-binding domains were used in experiments on the regulation of gene transcription and for studying the epigenomes and behavior of chromosomal loci in the cell cycle.

This review describes in detail the possibilities in the construction, implementation, and analysis of the TALEN and CRISPR/Cas9 function using examples of various model systems, as well as the complexities and problems associated with the use of these genome editing tools.

## NATURAL BACTERIAL TALE AND CRISPR/CAS SYSTEMS AS THE BASIS FOR THE DEVELOPMENT OF NEW TOOLS FOR EUKARYOTIC GENOME EDITING

**TALEN**

In 2011, *Nature Methods *named the methods of precise genome editing, including the TALEN system, method of the year [9]. The history of this system’s development is associated with the study of bacteria of the *Xanthomonas *genus. These bacteria are pathogens of crop plants, such as rice, pepper, and tomato; and they cause significant economic damage to agriculture, which was the motivate for their thorough study. The bacteria were found to secrete effector proteins (transcription activator-like effectors, TALEs) to the cytoplasm of plant cells, which affect processes in the plant cell and increase its susceptibility to the pathogen. Further investigation of the effector protein action mechanisms revealed that they are capable of DNA binding and activating the expression of their target genes via mimicking the eukaryotic transcription factors.

TALE proteins are composed of a central domain responsible for DNA binding, a nuclear localization signal, and a domain that activates the target gene transcription [10]. The capability of these proteins to bind to DNA was first described in 2007 [11], and just a year later two groups of researchers deciphered the code for recognition of the target DNA by TALE proteins [12, 13]. The DNA-binding domain was demonstrated to consist of monomers, each of them binds one nucleotide in the target nucleotide sequence. Monomers are tandem repeats of 34 amino acid residues, two of which are located at positions 12 and 13 and are highly variable (repeat variable diresidue, RVD), and it is they that are responsible for the recognition of a specific nucleotide. This code is degenerate; some RVDs can bind to several nucleotides with different efficiencies. Before the 5’-end of a sequence bound by a TALE monomer, the target DNA molecule always contains the same nucleo tide, thymidine, that affects the binding efficiency [14]. The last tandem repeat that binds a nucleotide at the 3’-end of the recognition site consists only of 20 amino acid residues and therefore is called a half-repeat.

After deciphering the code of DNA recognition by TALE proteins, which attracted the attention of researchers across the world due to its simplicity (one monomer – one nucleotide), the first studies on the construction of chimeric TALEN nucleases were launched. For that purpose, the sequence encoding the DNA-binding domain of TALE was inserted into a plasmid vector previously used for creating ZFN [15]. This resulted in the generation of genetic constructs expressing artificial chimeric nucleases that contain the DNA-binding domain and the catalytic domain of restriction endonuclease FokI. This system allow ones, by combining monomers of the DNA-binding domain with different RVDs, to construct artificial nucleases, the target of which can be any nucleotide sequence. Most studies use monomers containing RVDs such as Asn and Ile (NI), Asn and Gly (NG), two Asn (NN), and His and Asp (HD) for binding the nucleotides A, T, G, and C, respectively. Since the NN RVD can bind both G and A, a number of studies was performed to find monomers that will be more specific. It has been shown that the use of NH or NK monomers for more specific binding of guanine reduces the risk of off-target effects [16, 17]. The first amino acid residue in the RVD (H and N) was found not to be directly involved in the binding of a nucleotide, but to be responsible for stabilizing the spatial conformation. The second amino acid residue interacts with a nucleotide, with the nature of this interaction being different: D and N form hydrogen bonds with nitrogenous bases, and I and G bind target nucleotides through van der Waals forces [18].

**Fig. 1:**
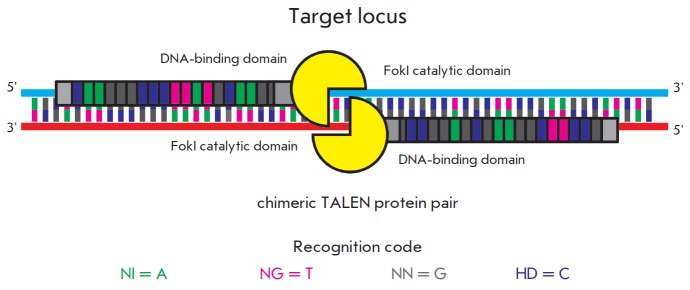
A scheme for introducing a double-strand break using chimeric TALEN proteins. One monomer of the DNA-binding protein domain recognizes one nucleotide of a target DNA sequence. Two amino acid residues in the monomer are responsible for binding. The recognition code (single-letter notation is used to designate amino acid residues) is provided. Recognition sites are located on the opposite DNA strands at a distance sufficient for dimerization of the FokI catalytic domains. Dimerized FokI introduces a double-strand break into DNA

An artificial DNA-binding domain is inserted into a genetic construct comprising a nuclear localization signal, half-repeat, N-terminal domain, and the FokI catalytic domain. TALENs work as pairs and their bindings sites are chosen sothat they are located on opposite DNA strands and are separated by a small fragment (12–25 bp), a spacer sequence. Once in the nucleus, artificial nucleases bind to target sites: the FokI domains located at the C-termini of a chimeric protein dimerize to cause a double-strand break in a spacer sequence (*Fig. 1*).

In theory, a double-strand break can be introduced in any region of the genome with known recognition sites of the DNA-binding domains using artificial TALEN nucleases. The only limitation to the selection of TALEN nuclease sites is the need for T before the 5’- end of the target sequence. However, site selection may be made in most cases by varying the spacer sequence length. The W232 residue in the N-terminal region of the DNA-binding domain was demonstrated to interact with 5’-T, affecting the efficiency of TALEN binding to the target site [19]. However, this limitation can also be overcome by selection of mutant variants of the TALEN N-terminal domain that are capable of binding to A, G, or C [14].

**CRISPR/Cas**

**Fig. 2:**
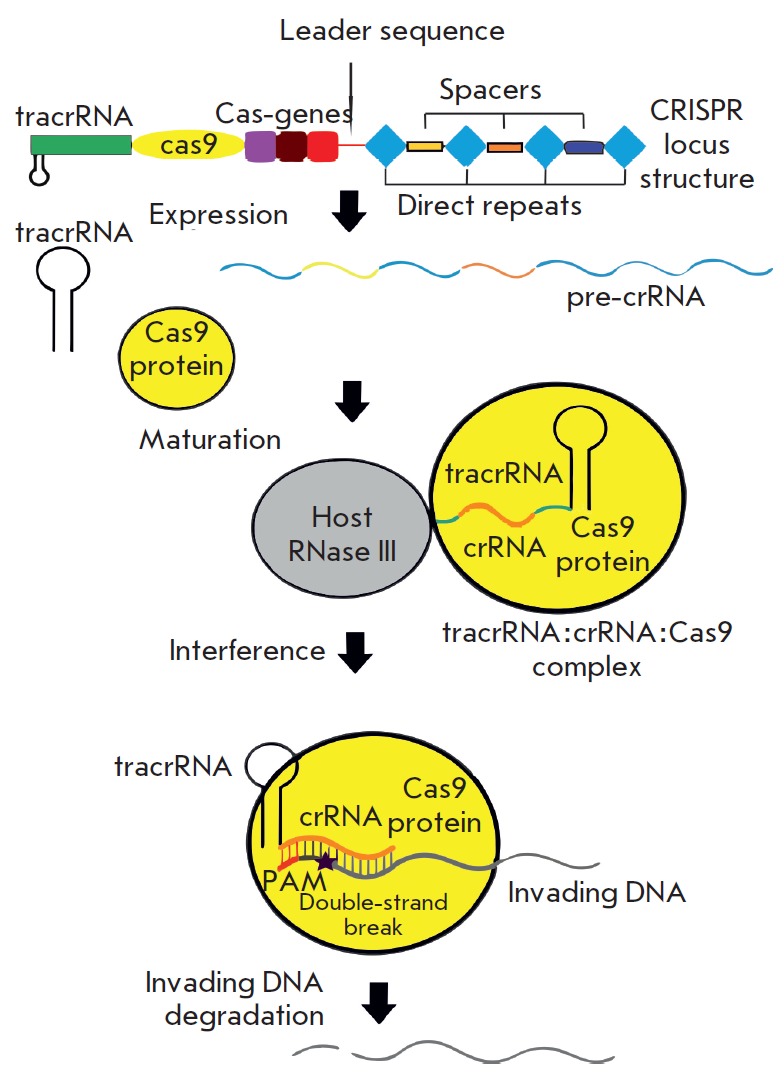
A mechanism of CRISPR/Cas9 action in bacterial cells (see the text for details)

About two years later after the discovery of the chimeric TALEN proteins, another genome editing system, CRISPR, elements of which are non-coding RNAs and Cas proteins (CR ISPR associated), was developed and started to be extensively used. In contrast to the chimeric TALEN proteins, recognition by the CRISPR/ Cas system is carried out via the complementary interaction between a non-coding RNA and the target site DNA. In this case, a complex of non-coding RNA and Cas proteins,which have nuclease activity, is formed. As early as 1987, mysterious repeats were discovered in some bacterial genes [20], the functions of which remained unknown for nearly 20 years. Sequencing of bacterial genomes revealed similar nucleotide sequences in the genome of many microorganisms that have the characteristic structure: short regions of the unique DNA (spacers) are separated from each other by short palindromic repeats (*Fig. 2*). Due to this feature, they received the name CRISPR (see abbreviations). Furthermore, these CRISPR cassettes are located in close proximity to the *cas *genes (CRISP associated), the protein products of which have helicase and nuclease activity [21]. In 2005, three independent groups of bioinformaticians reported that the spacer DNA is often homologous to the DNA of many phages and plasmids [22-24]. Furthermore, in 2007, it was shown that *Streptococcus thermophilus *cells bearing in the CRISPR locus a spacer that is complementary to a bacteriophage genomic DNA fragment become resistant to the phage [25]. Thus, it became apparent that the CRISPR/Cas system is the unique mechanism providing microorganisms protection against foreign DNA penetration and acting along with the restriction-modification system as a limiter of the horizontal transfer of genetic information.

The CRISPR systems are widespread in prokaryotes: they are found in 87% of archaea and 48% of eubacteria [26]. This is why different species are widely varied both in the number of CRISPR cassettes in the genome (1-18) and in the number (60, on average) and size of repeats (23–37 bp, on average), as well as in the number and size of spacers (17–84 bp). Yet, the length of spacers and repeats in one cassette is constant and repeat sequences are almost identical [27].

The protection mechanism includes three main stages (*Fig. 2*). At the first stage, adaptation, a small fragment of foreign DNA that entered a bacterial cell is inserted into the CRISPR locus of the host genome, forming a new spacer. In the viral genome, this fragment is present as a protospacer that is complementary to the spacer and flanked by a short (2–5 bp), conserved sequence called PAM (protospacer adjacent motif) [28, 29]. The new spacer is always inserted on the AT-rich side of the leader sequence located before a CRISPR cassette that also contains promoter elements and landing sites for regulatory proteins [30, 31]. Apparently, this is the way the targets of most of the CRISPR/Cas systems are formed.

At the second stage, transcription, the entire CRISPR locus is transcribed into a long pre-crRNA (poly-spacer precursor crRNA) (*Fig. 2*). The processing of an immature transcript into mature crRNA in most of the CRISPR/Cas systems is implemented by Cas6 endonuclease [32-36]. Short crRNAs (CRISPR RNA) of 39–45 nucleotides contain one spacer sequence, and their ends contain repeats involved in the formation of the stem loop structure: the last eight nucleotides of the repeat with a hydroxyl group at the 5’-end form the stem, and the hairpin structure with 2’, 3’-cyclic phosphate forms the loop at the 3’-end [37, 38].

The third stage, the interference of foreign DNA or RNA, is provided by the interaction between crRNA and a complex of Cas proteins; crRNA recognizes complementarily the protospacer sequence, and Cas proteins provide its degradation (*Fig. 2*).

For target DNA degradation by the effector complex, any interaction between the complementary nucleotides of crRNA and target DNA at positions –2, –3, and –4 (if the first protospacer base is taken as +1) should be avoided [39]. Apparently, complementary interactions between crRN A and the target DNA at these positions disrupt the effector complex formation, which prevents cleavage of genomic DNA and its subsequent degradation.

Long-term co-evolution of viruses and their hosts has led to the formation of viral protection mechanisms against the CRISPR interference [40], which explains a wide variety of the CRISPR/Cas systems in bacteria and archaea. Bioinformatic studies subdivide all CRISPR/Cas systems into three main types (I–III) and, at least, 10 subtypes [21, 27, 41]. Among these, the type II-A CRISPR/Cas system isolated from the *S. pyogenes* pathogen is currently the one used most widely in genomic engineering. A minimum set of the *cas *genes was found in this bacterium [27, 41]. One polyfunctional Cas9 protein performs both the processing of precrRNA and the interference of foreign DNA [42]. The crRNA processing also depends on a small non-coding RNA, tracrRNA (trans-activating crRNA). tracrRNA molecules bind complementarily to repeat sequences in pre-crRN A, forming a duplex, while one of the ribonucleases of the host cell, RNase III, cuts the duplex in the presence of Cas9 to form mature crRNA containing a 20 nucleotide spacer sequence at the 5’-end. Cas9 makes a double-strand break in the target locus in the presence of Mg^2+^ ions, with the HNH nuclease domain of the enzyme cutting the DNA strand complementary to crRNA, and the RuvC domain cutting the non-complementary strand [43]. The target DNA for Cas9 of *S. pyogenes *should necessarily contain 5’-NGG-3’ PAM [43, 44], three nucleotides from which cleavage occurs. In *S. thermophilus *and *Neisseria meningitides*, targets for type II Cas9 have a different consensus (5’-NGGNG- 3’ and 5’-NNNNGATT-3’, respectively).

## GENOMIC ENGINEERING USING TALENS AND CRISPR/CAS9

**Fig. 3:**
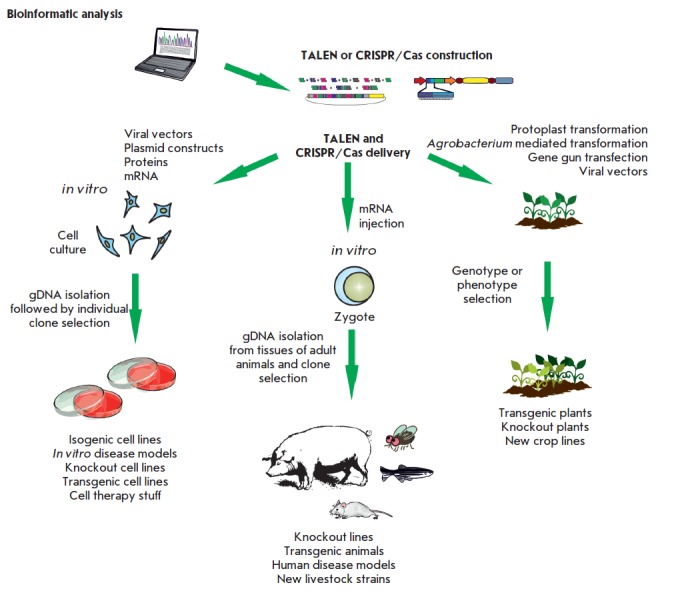
A general scheme of the strategy for using the TALEN and CRISPR/Cas systems in genomic engineering

The general strategy in genomic engineering using site-specific nucleases comprises four main stages (*Fig. 3*):

1. Selection of a target nucleotide sequence in the genome;

2. Generation of a nuclease construct directed at the selected target;

3. Delivery of this construct to the cell nucleus; and

4. Analysis of produced mutations.

**Selection of a target nucleotide sequence in the genome**

An important aspect of working with the TALEN and CRISPR/Cas9 systems is careful selection of sites for the specific introduction of a double-strand break. The need for a preliminary bioinformatic analysis is explained by the possibility for off-target effects – introducing non-specific double-strand breaks into the genome. When selecting desired sites, regions of repeated sequences, as well as regions with a high homology to other genome regions, should be avoided.

The off-target effects, when using the chimeric TALEN protein system, arise for several reasons. First, these are differences in the binding efficiency of RVD and specific nucleotides. HD and NN monomers form strong hydrogen bonds with nucleotides, while NG and NI form weak hydrogen bonds. This causes a possible binding of the DNA recognition domain to sites that differ from the target sites in a few nucleotides. Second, the degeneracy of the code for the binding of nucleotides by monomers may lead to, for example, interaction between NG and A. Third, dimerization of the FokI domains of two nucleases with identical DNAbinding domains (formation of homodimers) is possible. This issue has been resolved in a number of studies by producing TALENs that contain the FokI domains acting as obligate heterodimers. Finally, the possible offtarget effects may result from the fact that the size of the spacer DNA between the nuclease recognition sites is not fixed. This property makes it possible to introduce double-strand breaks during the binding of nucleases to off-target sites located at a distance sufficient for the dimerization of the FokI domains [45].

Since Cas9 nuclease of *S. pyogenes *needs the obligatory presence of the PAM with the 5’-NGG-3’ consensus, though it is not much, but it limits selection of a target. In particular, target sites in the human genome are located in every 8–2 bp [46, 47]. One of the main drawbacks of the CRISPR/Cas9 system is a relatively high probability of off-target mutations. A number of studies carried out *in vitro *[43], in bacteria [48], and in human cells [46] have demonstrated that some single nucleotide substitutions in the 20-nucleotide spacer region of sgRNA (single-guide RNA) may lead to a significant reduction in the activity of CRISPR/Cas9, especially if these substitutions are located in the last 10–2 nucleotides of the 3’-end of this region of sgRNA [49]. At the same time, substitutions at the 5’-end of sgRNA have actually no effect on the system’s activity [43, 46, 48]. However, cases are known when some single- and dinucleotide substitutions at the 3’-end of sgRNA do not affect the CRISPR/Cas9 system’s activity and, instead, inhibit its action, if they are located at the 5’-end [49]. In general, the off-target effect is determined by the position of substitutions, when 8–2 bp at the 3’-end of the guide sequence are less important for Cas9 than the 5’-end nucleotides; by the number of substitutions, which should not be more than three; by features of the very target site; by the concentration of introduced Cas9 and sgRNA [46-49]. The search for and development of methods based on the use of Cas9 orthologs, the activity of which needs a PAM with a more complex consensus sequence, will overcome these drawbacks. For example, type II CRISPR/Cas of *N. meningitidis *recognizes the PAM with the 5’-NNNNGATT-3’ consensus, which certainly limits the choice of a target but may increase the specificity.

In order to increase the specificity of genome editing based on the CRISPR/Cas system, two Cas9 nickases with a pair of sgRNAs are used [50, 51] by analogy with pairs of ZFNs and TALENs, which cause breaks in DNA only under the action of two independent proteins with the FokI domains. Mutations in one of the catalytically active domains (D10A in HNH and H840A in RuvC) convert Cas9 nuclease into DNA nickase [43, 46, 52]. If cleavage of both DNA strands by a pair of Cas9 nickases leads to the formation of site-specific doublestrand breaks that are repaired via non-homologous end joining (NHEJ), individual single-strand damages are primarily repaired via highly accurate base excision repair (BER) [53]. The use of two Cas9 nickases with a sgRNA couple was demonstrated to provide a significant reduction in the production of off-target mutations, with the yield of target mutations generally corresponding to that for the use of nuclease [50, 51].

The mentioned properties of target site recognition by the CRISPR/Cas9 and TALEN systems were taken into account when developing computer algorithms that search for these sites. Currently, on-line software is available that was developed by different teams and designated for the selection of potential sites for the TALEN [54-59] and CRISPR/Cas9 [47, 60-62] systems, as well as for the detection of possible off-target effects.

**Generation of genetic constructs expressing CRISPR and TALEN**

*TALEN*. The DNA-binding domain consists of almost identical repeats: so there are certain technical difficulties in creating genetic constructs expressing TALENs. A number of methods have been suggested that enable to construct TALE DNA-binding domains consisting of 20–30 or even more monomers. One of the strategies is based on standard DNA cloning using hydrolysis by type II DNA restriction endonucleases and ligation: REAL (REstriction and Ligation [63]). At the first stage, a library of monomers is generated that are introduced with endonuclease restriction sites at the 5’- and 3’-termini. After DNA hydrolysis, pair-wise ligation is carried out resulting in the formation of dimers (N1N2, N3N4, N2k-1N2k) that are then combined in tetramers and so forth. In this case, the correct sequence is achieved by the use of various restriction endonucleases. This technique is rather difficult and time consuming, because at each stage, the reaction products should be purified, and the correct orientation should be confirmed. To accelerate this process, a library of 376 members was generated that consists of mono- , di- , tri-, and tetramers (REAL-Fast, [64]).

**Fig. 4:**
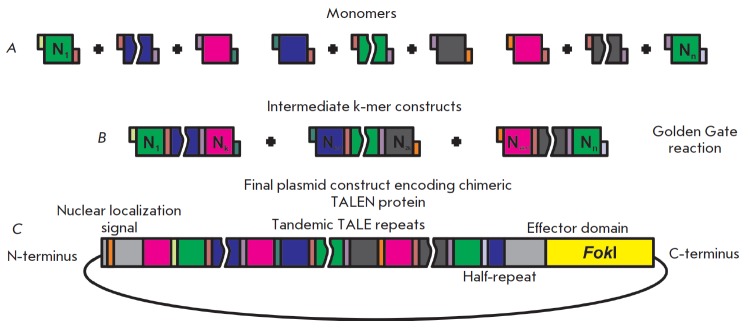
A scheme of the modular hierarchical ligation strategy based on the Golden Gate cloning system to generate genetic constructs expressing chimeric TALEN proteins. A – at the first stage, a library of monomers is generated, which is a kind of a “construction kit” comprising a set of parts. These parts are amplified sequences of monomers with specific oligonucleotide primers. The primers are selected in such a way that hydrolysis by type IIS restriction endonucleases results in the formation of sticky ends, which define the monomer position in a final construct. B – a single Golden Gate reaction enables simultaneous ligation of multiple monomers, which results in intermediate k-mer constructs. C – at the last stage, the Golden Gate reaction is carried out, resulting in restriction and ligation of several intermediate k-mer constructs and the backbone plasmid containing the remaining TALEN elements

To increase the efficiency and to accelerate the assembly process, the Golden Gate reaction is used [65, 66], which is simultaneous ligation and hydrolysis by restriction endonucleases in the same reaction mixture (*Fig. 4*). In the Golden Gate reaction, type IIS restriction endonucleases are used that hydrolyze DNA at a fixed distance from the recognition site, for example, BsmBI or BsaI. Therefore, the “scarless” assembly occurs during ligation, because restriction endonucleases “cut off” their own site from a monomer, and the ligation product is not subjected to restriction. A library that contains different variants of the all four monomers, corresponding to the various positions (e.g., 1 to 20) in the future DNA-binding domain, is generated by amplifying sequences of monomers (NI, HD, NG, and NN) with different oligonucleotide primers. Treatment of these monomers with IIS restriction endonucleases produces sticky ends complementary to the sticky ends of neighbor monomers. In a single reaction, several monomers can be simultaneously ligated; for example, four [67] and six [68]. Next, using the Golden Gate reaction again, it is possible to ligate several tetra- or hexamers and to clone a complete sequence into a plasmid vector containing the 3’-half-repeat and FokI catalytic domain.

In order to reduce the time needed to develop genetic constructs expressing TALEN, a method was proposed that enables the exclusion of DNA ligation and, accordingly, steps related to verification of its results. The selected DNA-binding domain is assembled from monomers with long, specific, single-strand ends (10–30 nucleotides). Upon mixing several monomers, annealing of complementary single-strand ends occurs, whereby the monomers are arranged into a desired sequence. Then, *E. coli *cells are transformed with the resulting mixture and ligation occurs already in bacteria with involvement of their own enzymes [69].

These methods for developing genetic constructs expressing TALENs are relatively simple, and, according to various estimates, their implementation takes 1–2 weeks, if appropriate reagents are available. In addition to the simplicity and efficiency, this technology is also easily accessible; currently at the Addgene Depository (http://www.addgene.org/TALEN/), kits for the construction of TALENs developed by different groups of authors [64, 68-71] can be purchased and used in the laboratory.

Also, there are available systems for the automated high-performance production of constructs expressing TALENs nucleases. For example, a commercial platform from Cellestis Bioresearch enables one to generate up to 7,200 of these constructs annually. Three methods based on the use of solid phase surfaces have been described in the scientific literature [72-74]. These methods avoid an analysis of intermediate constructs, their purification by extraction from the gel, and other stages, which makes these methods suitable for automated production and accelerates the process. The idea behind these methods is to use streptavidincoated magnetic particles with attached biotinylated double-strand DNA adapters. Sequential alternation of the phases of DNA hydrolysis by restriction endonucleases and ligation is used to extend a sequence of monomers that is connected via an adapter with the magnetic particle. The reaction products are purified by means of washing buffers on a magnetic plate. In this case, by-products and reaction components are washed away and the target product is retained in a test-tube (or well) due to the attraction between the magnetic particles and the plate. At the end, restriction endonucleases are used to cleave the links between the biotinylated adapter and the synthesized sequence of monomers of the DNA-binding domain of TALEN. The sequence is then cloned into a plasmid vector by means of DNA ligation. This method allows one to quickly and efficiently synthesize in parallel genetic constructs in 96-well plates using multichannel pipettes or robotic pipetting stations.

*CRISPR/Cas9. *It was demonstrated that for cleavage of DNA *in vitro *[43, 52] and in bacterial cells [42] using CRISPR/Cas9, the following components are necessary and sufficient: non-coding RNAs (tracrRNA and pre-crRNA), RNase III, and the Cas9 protein. The use of this system in mammalian cells exhibits several features.

First, SpCas9 nuclease (Cas9 of *S. pyogenes*) should be adapted for adequate transcription in high eukaryotic cells, in particular codon-optimized, and attachment of nuclear localization signals (NLS) is necessary to provide a nuclear compartmentalization; two NLS are sufficient for effective guiding of Cas9 to the nucleus [46].

Second, maturation of pre-crRNA in eukaryotic cells does not require the introduction of exogenous RNase III, since this function is successfully performed by its own cellular RNases [75-77].

**Fig. 5:**
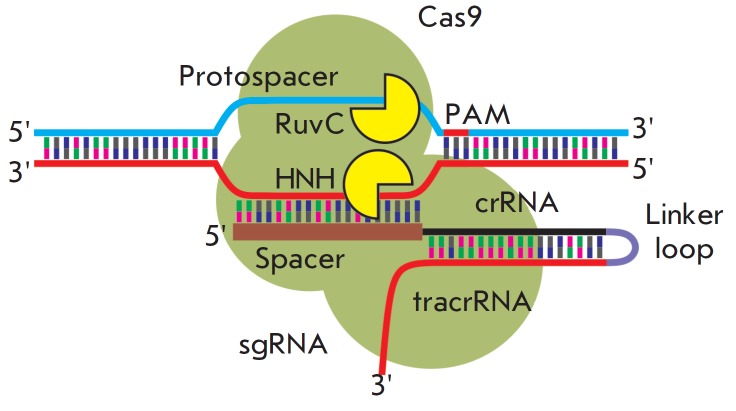
Single chimeric sgRNA to introduce double-stranded breaks into the target loci. A complex of sgRNA and Cas9 is capable of introducing double-strand breaks into selected DNA sites. SgRNA is an artificial construct consisting of elements of the CRISPR/Cas9 system (crRNA and tracrRNA) combined into a single RNA molecule. A protospacer is a site that is recognized by the CRISPR/ Cas9 system. A spacer is a sequence in sgRNA that is responsible for complementary binding to the target site. RuvC and NHN are catalytic domains causing breaks at the target site of the DNA chain. PAM is a short motif (NGG in the case of CRISPR/Cas9) whose presence at the 3’-end of the protospacer is required for introducing a break

Third, instead of two non-coding RN As, single chimeric sgRN A is often introduced, in which mature crRN A is fused with a part of the tracrRNA through the synthetic “stem-loop” structure to simulate the natural crRNA-tracrRNA duplex [43] (*Fig. 5*). To transcript sgRN A, an appropriate promoter is required: for example, the RNA polymerase III U6 promoter.

**Fig. 6:**
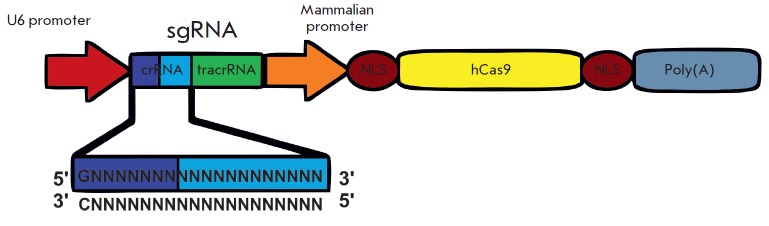
A scheme of a genetic construct expressing CRISPR/Cas system elements. hCas9 is the Cas9 protein sequence optimized for expression in eukaryotic cells. sgRNA is a single chimeric RNA containing the parts of crRNA and tracrRNA necessary for the activity. NLS is the nuclear localization signal, which provides penetration of the construct into the nucleus. Poly (A) is the polyadenylation signal

Basic plasmid constructs containing the elements necessary for CRISPR/Cas9 activity were produced in the Feng Zhang’s laboratory. The pX260/pX334 plasmids contain three expression cassettes: Cas9 nuclease/ nickase, CRISPR mRNA, and tracrRNA (*Fig. 6*). To change the target sequence, this construct only needs cutting off the original 30 nucleotide guide sequence flanked by BbsI sites and replacing it with an artificially synthesized one. To this effect, 30-mer oligonucleotides complementary to the target sequence and containing the appropriate sticky ends are melted together and ligated to the plasmid.

pX330/pX335 plasmids contain two expression cassettes: Cas9 nuclease/nickase and chimeric sgRNA comprising 85-nucleotide tracrRN A. The principle of changing the guide sequence is the same, but the sequence length is shorter – 20 nucleotides –and the 20th position should be occupied by guanine, because the U6 promoter used in this case comprises this base at the transcription start point. Furthermore, these plasmids can be inserted with additional elements, such as the 2A-GFP or 2A-Puro sites, for subsequent selection of cells bearing the plasmid.

**Delivery of constructs expressing CRISPR/Cas9 components**

To transform human, mouse, and other cell cultures, plasmids providing extensive production of Cas9 nuclease and sgRNA *in vitro *are more often used [46, 78-80]. To transform the whole organism, a method based on microinjection of *cas9 *mRNA and sgRNA into single- celled embryos was developed [81-83]. This method is widely used in mouse, zebrafish (*Danio rerio*), and drosophila. For large-scale genome-wide knockout using large sgRNA libraries, lentiviral vectors are employed [84, 85]. In plants, which have cells with a thick cellular wall, the method of protoplast-plasmid transformation in cell cultures [86, 87], as well as agroinfiltration using *Agrobacterium tumefaciens *[88, 89], is widely used.

**Analysis of mutations caused by CRISPR/Cas9 and TALEN**

**Fig. 7:**
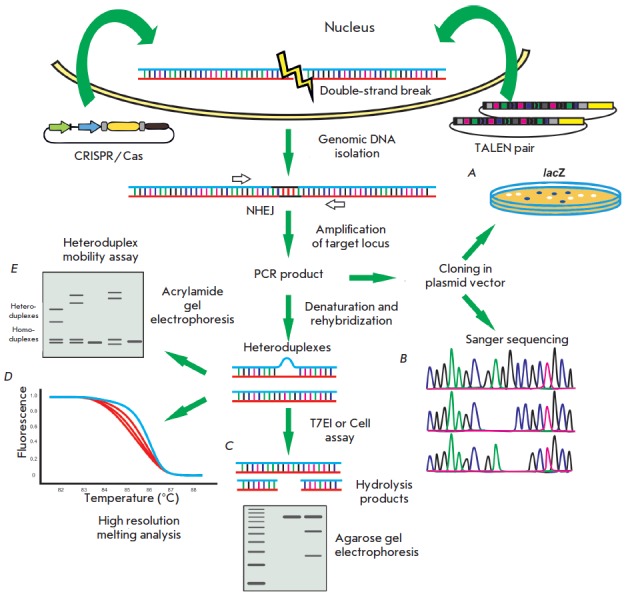
A scheme of various analyses to identify and determine the efficiency of double-strand break introduction caused by the TALEN and CRISPR/Cas systems. First, constructs encoding CRISPR/Cas9 or TALEN are delivered into cells. In cells, double-strand breaks occur in the target loci that are repaired by nonhomologous end joining (NHEJ). This results in the formation of insertions or deletions. Next, the target locus is amplified by PCR. PCR products are analyzed by the following methods. A – a target segment is cloned into a plasmid vector. Impairment or, instead, recovery of the reading frame of the lacZ gene occurs due to the insertions or deletions. Based on the count of blue and white colonies after the transformation of E. coli, the efficiency of the CRISPR/Cas9 or TALEN systems is determined; B – after cloning into a plasmid vector and E. coli transformation, Sanger sequencing is performed. Clones containing insertions/deletions are counted, the efficiency is determined; C – after denaturation and re-hybridization of the PCR product, DNA heteroduplexes are formed; e.g., one strand is “wild type,” and the other contains a deletion. After treatment with enzymes that cut DNA in unpaired segments, samples are loaded onto a gel and electrophoresis is carried out. The hydrolysis products mean that the sample contained heteroduplexes; hence, a break appeared in double-strand genomic DNA under the action of CRISPR/Cas9 or TALEN; D – a high resolution melting analysis enables heteroduplex detection. Blue is the control samples, red is the samples containing heteroduplexes; E – unpaired DNA regions reduce the heteroduplex mobility in a 15% polyacrylamide gel. After gel electrophoresis, bands corresponding to homo- and heteroduplexes can be observed

Due to the activity of CRISPR/Cas9 or TALENs systems, a double-strand break is introduced into eukaryotic DNA in the region of the CRISPR/Cas9 protospacer or spacer sequence separating the TALEN recognition sites (*Fig. 7*). In the absence of a homologous donor DNA, the double-strand break is repaired by nonhomologous end joining. During this process, errors occur and small insertions or deletions happen at a high frequency in the joining region [90]. A number of techniques based on the detection of such changes in a target DNA have been developed to study the activity of artificial nucleases in eukaryotic cells (*Fig. 7*).

A method based on TOPO cloning allows one to study the nucleotide sequences of mutant alleles resulting from nonhomologous DNA end joining as well as highly accurate quantification of the efficiency of artificial nucleases (*Fig. 7*). Eukaryotic cells are treated with artificial nucleases, then genomic DNA is isolated, and the DNA segment containing a nuclease recognition site is amplified by PCR . The PCR products are cloned in a plasmid vector, followed by sequencing of the clones produced after the transformation to *E. coli* cells [72]. Based on this, the variety of the generated mutations and their frequency are determined. Furthermore, if the cells treated with artificial nucleases are used to produce clonal populations, then lines carrying certain mutations may be selected after sequencing. For example, based on a selection of clones with a deletion of a certain size, cell lines were produced in which the reading frame impaired by the Duchenne muscular dystrophy mutation was restored [91].

The artificial nuclease activity is analyzed using enzymes that cleave the phosphodiester bonds in unpaired DNA segments (*Fig. 7*). Amplification of a segment selected as a target for artificial nucleases produces a mixture of DNA molecules, the nucleotide sequences of which are different due to the insertions or deletions that occurred during nonhomologous end joining. Denaturation followed by re-hybridization of a PCR product results in the formation of heteroduplexes containing loops in unpaired segments. After rehybridization, PCR products are treated with enzymes such as phage T7 endonuclease I [92] or nucleases of the CELI family [93], and then the resultant fragments are separated by electrophoresis. Detection of hydrolysis products indicates that a PCR product mixture contains fragments with insertions or deletions resulting from nonhomologous end joining. The efficiency of artificial nucleases may be estimated by the ratio of intensity of the main product and the fragments produced during the hydrolysis, but this is an inaccurate estimate [92].

The properties of the resulting heteroduplexes are different from those of homoduplexes. One of these differences is a change in the melting curve profile that can be detected using the high resolution melting analysis (HRMA) (*Fig. 7*). A short segment (100–300 bp) containing a double-strand break site is amplified using real-time PCR with fluorescent intercalating dyes. Then, after denaturation and rehybridization, HRMA is performed. Based on a comparison of the control and test samples, changes in the melting curve profile and, hence, changes in the nucleotide sequences resulting from nonhomologous end joining can be determined [94]. This analysis is sensitive and simple, but this is a qualitative method that does not allow one to estimate accurately the efficiency of artificial nucleases, as well as the nature of DNA changes.

Another method to determine whether a doublestrand break was introduced into a target site is the analysis of the electrophoretic mobility of heteroduplexes. Unpaired segments of the single-strand DNA that form loops in heteroduplexes reduce their mobility in a 15% polyacrylamide gel compared to that of homoduplexes. This property makes it is possible not only to determine whether a double-strand break occurred, but also to evaluate the variety of generated mutations as well as to genotype different clones, because different size deletions or insertions change the heteroduplex mobility in different ways. In this case, the mobility profile for lines containing the same mutation is also identical [95].

The efficiency of artificial nucleases can be quantified and compared using methods based on genetic reporter constructs containing genes of luminescent proteins. In this case, single-strand annealing (SSA) is used, which is one of the ways used to repair doublestrand breaks in the genome of eukaryotes. If a doublestrand break occurs between two direct repeats, then annealing of the complementary sequences flanking the break occurs via SSA. Then, the nonhomologous regions are hydrolyzed by specific nucleases and the synthesis and ligation of new DNA occur in singlestrand segments. The sequence between direct repeats where a double-strand break occurred is always deleted, and one sequence remains instead of two repeated sequences. This process is used to restore a reporter gene; e.g., the luciferase gene. After a double-strand break introduced into the target sequence cloned into a plasmid vector between two repeat elements of the reporter gene, the reporter function is restored by means of SSA. Therefore, the efficiency of artificial nucleases can be quantified by the level of luminescence. In this case, reporter constructs are transfected into eukaryotic cells such as HEK293 lines or some yeast strains. The disadvantage of this method is that it does not take into account the genomic environment in which the taget site is located; so, its results may not correlate with the results obtained when working with target sites in the genome [96].

Japanese scientists have developed a method of analysis based on the impairment/restoration of the* lacZα *gene function (*Fig. 7*). For this purpose, the site designated for introducing a double-strand break is cloned into the *lacZα *gene. In this case, oligonucleotide primers are selected in such a way that the wild type target site impairs (1) or preserves (2) the reading frame. If a double-strand break occurred in the site that was repaired by nonhomologous end joining, then in the first case, after cloning the reading frame will be restored in the one-third constructions due to deletions or insertions. Accordingly, after the transformation of* E. coli *cells with the produced constructs, a fraction of the colonies will be blue in color. In the second case, the reading frame will be impaired in the two-third constructs due to mutations caused by artificial nucleases. The colonies with these genetic constructs will be white in color. The efficiency of artificial nucleases can then be determined by simply counting the fraction of blue or white colonies in the first and second cases, respectively [97].

## APPLICATION OF CRISPR/CAS9 AND TALEN SYSTEMS

Nuclease makes double-strand breaks in a target site that are repaired by the cell through one of two possible mechanisms:

Nonhomologous end joining, when errors occur that result in indel type (insertions, deletions) mutations in the target locus.

Homologous recombination, in which an intact homolog serves as a template to restore the original DNA structure; this is quite a rare event in the cell, but the use of CRISPR/Cas9 and TALENs increases the probability of homologous recombination by several orders of magnitude. If CRISPR/Cas9 components are added with artificially synthesized DNA showing homology with a nucleotide sequence at the break, then it may serve as a template for another way to repair DNA, homology- directed repair (HDR), in which a small piece of an artificial template is introduced into the target locus. As such a template, two types of constructs are most often used: single-strand oligonucleotides and plasmid vectors. In the first case, oligonucleotides homologous to the site for double strand break introduction are artificially synthesized; the optimum oliginucleotide length is about 90 nucleotides [98]. These oligonucleotides may be slightly different from the target site. When plasmid vectors are used as donor molecules for recombination, sufficiently long homology arms are cloned in them (500 to several thousand base pairs). These homology arms can flank additional elements such as reporter genes, antibiotic resistance genes, and so forth. Besides transgenesis HDR can be used to alter the genome via original nucleotide sequence replacing: synonymous substitutions can be generated to provide a new restriction site or a mutant allele, for example, which causes some hereditary disease, can be replaced with wild type allele. (genetic correction)However, HDR occurs vigorously only in dividing cells and its efficiency is highly depends on the cell type, stage of life, as well as the target locus of the genome and template itself [99].

Therefore, the following mutations can be produced using site-specific nucleases:

• non-homologous end joining in the absence of a donor plasmid mediates deletions or insertions of several nucleotides in the target site and, as one of the results, knockout due to reading frame mutations and stop codon formation [100];

• in the presence of double-strand oligonucleotides or a donor plasmid, DNA fragments of more than 14 kb can be inserted through nonhomologous end-joiningmediated ligation [101, 102];

• simultaneous introduction of several doublestrand breaks may lead to deletions, inversions, or translocations of the DNA regions located between these breaks [46, 103];

• homologous recombination in the presence of a donor plasmid with homology arms flanking the inserted fragment [104], a linear donor sequence with homology of less than 50 bp [105], or an oligonucleotide [103] leads to insertion of one or more transgenes for the correction or replacement of existing genes. Currently, these methods are extensively used in basic and applied research. In this case, genome editing is possible both *in vitro *upon delivery of TALEN or CRISPR/Cas elements to cell cultures and *in vivo *by mRNA injections into zygotes (*Fig. 3*).

Currently, these methods are extensively used in basic and applied research. In this case, genome editing is possible both *in vitro *upon delivery of TALEN or CRISPR/Cas elements to cell cultures and *in vivo *by mRNA injections into zygotes (*Fig. 3*).

***In vitro *genome editing**

The HEK 293T/HEK 293FT cell lines are commonly used to test the efficiency of the TALEN and CRISPR/ Cas systems in a human *in vitro *model, because they can be transfected easily by plasmids and are relatively simple to maintain, [46, 50, 68, 78, 106]. According to different authors, the level of targeted mutations and also homologous recombination with donor plasmids/ oligonucleotides varies widely, which probably depends not only on the method, but also on the cell line and the genomic target itself (*Table*). Cultured lines of induced pluripotent stem cells and human embryonic stem cells are of particular interest for regenerative medicine, the investigation of the structure and functioning of complex gene networks, the development of drug search systems, and a variety of other basic and biomedical studies.

Using the TALEN system, Ding *et al*. [71] introduced double-strand breaks and obtained human stem cell lines with mutations in various disease genes. In total, 15 genes were mutated and a comprehensive phenotype analysis of differentiated derivatives of stem cells with mutations in four of them (*APOB*, *SORT1*, *AKT2*, and *PLIN1*) was performed. New data on the role of these genes in the pathogenesis of diseases were obtained due to these cell models. For example, the *APOB* gene product was demonstrated to be necessary for the replication of the hepatitis C virus in human hepatocytes. Viral replication is greatly reduced in cells with a homozygous mutation in this gene. What is more, the E17K mutation in the *AKT2 *gene leads to a decrease in the glucose synthesis in human hepatocytes and an increase in the level of triglycerides in adipocytes.

In addition to the generation of the models required for developing approaches to the treatment of diseases, artificial nucleases may be used directly for therapeutic purposes. One of such trends is the therapy of chronic viral infections. TALEN s may be constructed to allow the introduction of mutations into the open reading frames of viruses such as HIV, hepatitis B, and herpes, which may be present in the body in a latent state and not be affected by therapy against replicating viruses [107, 108]. For example, the *C-C *chemokine receptor type 5 gene of T lymphocytes, whose mutations render a person resistant to HIV, can be modified using TALENs [100, 109].

With the use of a CRISPR/Cas9-based technology, isogenic human stem cells were generated [110], methods to correct a mutant cell phenotype are developed [111], and studies on the gene expression regulation [112-116], functional relationships between large groups of genes [84, 85], and imaging regions of the active genome regions in living cells [117] are conducted.

The development of panels of human isogenic pluripotent stem cells will implement modeling of hereditary and multifactorial diseases, screening of large drug libraries, as well as searching for new mutations involved in the pathological process. Currently, all these areas are under extensive study. For example, the CRISPR/Cas9 system was effectively used to generate a ICF syndrome model (ICF is the immunodeficiency, centromeric region instability and facial anomalies syndrome) using human-induced pluripotent stem cells. Homozygous mutations in the *DNMT3B *gene with a frequency of 63% were generated, with cells having the centromeric instability phenotype [110]. It seems particularly relevant to study severe neurodegenerative diseases such as Alzheimer’s, Parkinson’s, and various muscle atrophies.

Since Cas9 recognizes a particular target in the genome with the participation of a short guide sequence in sgRNA, currently, it is relatively easy to generate a large genome-wide library of oligonucleotides and, accordingly, sgRNAs. Furthermore, the use of lentiviruses, which are stably maintained in the genome and replicated together with genomic DNA, as a vector for the delivery of CRISPR/Cas9 components, has allowed one the develop a new GeCKO (Genome-scale CRISPR/ Cas9 knockout) technology [84]. A large sgRNA library allows researchers to turn off the transcription of many genes simultaneously and thereby identify the functional relationships among them, their role in certain life processes, or their involvement in the pathological process. For example, the genes necessary for the life activity of cancer cells (A375 cell line of human melanoma) and pluripotent stem cells (HUES62 line) were identified using a lentivirus library comprising 18,080 genes (three or four sgRNAs for each gene) [84]. The development of resistance to vemurafenib (PLX), which is a BRAF inhibitor of protein kinases in melanoma, was demonstrated to involve not only the *NF1 *and* MED12 *genes, but the *CUL3 *gene as well as the genes of the STAGA complex of histone-specific acetyltransferases:* TADA1 *and *TADA2 *[84]. Based on a lentiviral library comprising about 73,000 sgRNAs, the genes involved in the proliferation and cell cycle were studied using the HL60 and KBM7 tumor cells [83]. It was demonstrated that mutations resulting in the formation of nonfunctional products of four DNA mismatch repair (MMR) genes (*MSH2*, *MSH6*, *MLH1*, and *PMS2*) cause resistance to a nucleotide analog, 6-thioguanine, and therefore provide the cell proliferation. The activity of the genes *TOP2A*, *CDK6*, *BCR*, and *ABL1 *and the genes that encode ribosomal proteins was also studied.

Therefore, the use of CRISPR/Cas9 libraries allows one to perform functional screening of genomes, which may yield important information about the physiology and biochemistry of different types of cells and could help reveal the molecular mechanisms of disease development and identify potential targets for drug and gene therapy.

CRISPR/Cas9-based methods can be effectively used to edit the genomes of cultured stem cells. In particular, the use of genome-editing systems enables one to correct point mutations in the cells obtained from patients. The object of research in this case may be induced pluripotent stem cells and regional stem cell. In this case, both complex genetic constructs and singlestrand DNA oligonucleotides can be used as donor molecules [98].

An interesting example of this approach is a study in which correction of the *CFTR *locus (cystic fibrosis transmembrane conductor regulator) was performed in cultured intestinal stem cells derived from cystic fibrosis (CF) patients [111]. This approach enables the so-called organoids obtaining: functional multicellular structures with a corrected genome that are autologous with respect to the cell donor, which may be administered back to the patient. Certainly, this trend offers great opportunities for cell therapy of human diseases.

The controlled introduction of transgenes into the genome may be used in the case of functional correction of the genetic abnormalities associated with gene deletions or expression impairments that manifest themselves in a significant reduction in the level of gene products (protein or RNA). There are genome re considered safe. These are sites like AAVS1 that provide stable expression of the introduced transgene [118]. Thus, the TALEN and CRISPR/Cas systems can effectively be used in the functional genomics of cells for the generation of cell models of human diseases and for cell therapy.

***In vivo *genome editing**

In genetics, for many years of its existence, a number of model objects have been formed that are studied in most detail and used in most basic and applied research. Model organisms include, for example, yeast, nematode, drosophila, arabidopsis, zebrafish, laboratory mice, and rat. These and a number of other model organisms are extensively used to perform experiments on genomic engineering using the CRISPR/Cas9 and TALEN systems.

Various applications of CRISPR/Cas and modifications of the genome editing technology in the nematode* Caenorhabditis elegans *are presented in a number of studies [119-126]. By injection of a mRNA/Cas9 protein and *in vitro/in vivo *produced sgRNA in germline cells, stable targeted genome modifications were produced in adult animals in the next generation, including small insertions/deletions, larger chromosome deletions and rearrangements [119], and transgene introduction by homologous recombination with donor molecules [121, 123]. This method is widely used to study the processes of dosage compensation in nematode and to compare gene functions in related species of *C. elegans *and *C. briggsae *[122].

The fruit fly, *Drosophila melanogaster*, is among the most studied model objects. However, the production of new mutant alleles by homologous recombination still remains a very labor-intensive procedure [127-129]. Injection of *cas9 *mRNA and sgRNA into drosophila embryos provides double-strand breaks in the target loci of the genome, repair of which leads to the generation of insertion/deletion type mutations at a very high level (*Table*). Embryo injection produces mutations in both alleles of the target gene in all cells of a developing, and adult afterwards, insect; however, a certain percentage of mosaics emerges in this case [130-132]. These mutations are stably transmitted from generation to generation, which provides the possibility to generate new lines of flies [133]. Recently, an application was developed (http://www.flyrnai.org/crispr) that enables effective experiments planning for editing the drosophila genome. Therefore, the CRISPR/ Cas9 technology allows quick and efficient generation of mutations to further study the gene activity in *Drosophila*.

The zebrafish is currently a very popular object not only for basic research of the structural and functional relationships in the genome, but also for modeling of metabolic and neurodegenerative diseases in humans *in vivo *[134]. Various target and stably inheritable modifications were generated by injection of CRISPR/Cas9 components into zebrafish embryos (*Table*). In 2011, the international Zebrafish Mutation Project was launched to generate mutant alleles in each zebrafish protein-coding gene. All data are analyzed on the website http://www.sanger.ac.uk/Projects/D_rerio/ zmp. As of June 2013, mutant models of 46% of all protein-coding zebrafish genes have been generated.

**Table:** Genomic engineering using TALEN and CRISPR/Cas

Nuclease	Object	Gene	Objective	Reference
TALEN	Human cells (Homo sapiens)	ccr5, akt2, e17k, angptl3, apob, atgl, c6orf106, celsr2, cftr, ciita, foxo1, foxo3, gli1, glut4, hbb, hdac1, hdac2, hdac6, hmga2, hoxa13, hoxa9, hoxc13, hprt, il2rg, jak2, kras, linc00116, maoa, map2k4, mdm2, met, mlh1, msh2, mutyh, myc, mycl1, mycn, nbn, ncor1, ncor2, nlrc5, ntf3, pdgfra, pdgfrb, phf8, plin1, pms2, ppp1r12c (aavs1), ptch1, pten, rara, rbbp5, recql4, ret, runx1, sdhb, sdhc, sdhd, setdb1, sirt6, smad2, sort1, sox2, klf4ss18, suz12, tfe3, tp53, trib1, tsc2, ttn, vhl, xpa, xpc, abl1, alk, apc, atm, axin2, bax, bcl6, bmpr1a, brca1, brca2, cbx3, cbx8, ccnd1, cdc73, cdk4, cdh4, chd7, ctnnb1, cyld, ddb2, ercc2, ewsr1, ext1, ext2, ezh2, fanca, fancc, fancf, fancg, fes, fgfr1, fh, flcn, flt4, mstn, aavs2, oct4, pitx3	Knockout, insertion	[67, 68, 70–72, 74, 92, 176–179, 180]
	Yeast (Saccharomyces cerevisiae)	URA3, ADE2, LYS3	Knockout, insertion	[181]
	Nematode (Caenorhabditis elegans)	ben-1, tex-1, sdc-2	Knockout	[182]
	Drosophila (Drosophila melanogaster)	yellow, crhdr1, ponzr1, bmil, cdh5, dip2a, elmo1, epas1b, fh, golden, gria3, hey2, hif1ab, ikzf1, jak3, moesina, myod, phf6, ppp1cab, ryr1a, ryr3, scl6a3, tbx6, tnikb, th, fam46c, smad5	Knockout, insertion	[94, 183–187]
	Silkmoth (Bombyx mori)	blos2	Knockout	[188]
	Cricket (Gryllus bimaculatus)	lac2	Knockout	[189]
	Western clawed frog (Xenopus tropicalis)	ets1, foxd3, grp78/bip, hhex, noggin, ptf1a/p48, sox9, vpp1	Knockout	[190]
	Mouse (Mus musculus)	c9orf72, fus, lepr, pak1ip1, gpr55, rprm, fbxo6, smurf1, tmem74, wdr20a, dcaf13, fam73a, mlkl, mstn, pibf1, sepw1, rab38, zic2	Knockout, insertion	[179, 191–196]
	Rat (Rattus norvegicus)	bmpr2, IgM	Knockout	[197, 198]
	Pig (Sus scrofa)	amely, dmd, gdf8, ggta, ghdrhdr, il2rg, ldlr, rag2, rela (p65), sry	Knockout	[199]
	Cattle (Bos taurus)	acan, gdf8, ggta, mstn, prnp	Knockout	[179, 199]
	Arabidopsis (Arabidopsis thaliana)	adh1	Knockout	[70]
	Tobacco (Nicotiana benthamiana)	surA, surB, hax3	Knockout, insertion	[156, 157]
	False brome grass (Brachypodium distachyon)	aba1, cxk2, coi1, hta1, rht, sbp, smc6, spl	Knockout	[154]
	Rice (Oryza sativa)	avrxa7, pthxo3, badh2, ckx2, dep1, sd1	Knockout	[154, 155]
CRISPR/ Cas	Yeast (Saccharomyces cerevisiae)	CAN1, ADE2	Knockout, insertion	[200]
	Human cells (Homo sapiens)	dnmt3b-tdTomato, pou5f1(oct4), emx1, dyrk1a, grin2b, egfp, ccr5, c4bpb, pvalb, aavs, akt2, celsr2, ciita, glut4, linc00116, sort1, ldlr	Knockout, insertion	[46, 51, 78, 80, 201, 202]
	Nematode (Caenorhabditis elegans)	dpy-11, unc-4, ben-1, unc-36, daf-2, klp-12, lab-1, egfp, dpy-11, lin-5, rol-1, dpy-3, unc-1, dpy-13, unc-119, klp-12	Knockout, insertion	[119–124, 126]
	Drosophila (Drosophila melanogaster)	yellow, white, rosy, cg14251 (k81), cg3708cg17629 (kl-3), light	Knockout, insertion	[130–133]
	Danio rerio (Danio rerio)	etsrp, gata5, etsrp, gsk3b, apoea, fh, fh1, th1, rgs4, tia1l, tph1a, drd3, egfp, tyr, gol, mitfa, ddx19, sema3fb, dre-mir-126a, dre-mir-126b, dre-mir-17a-1–dre-mir-92a-1, dre-mir-17a-2–dre-mir-92a-2, fgd5, ensdarg00000070653, ensdarg00000076787, psmf1, dre-mir- 126a, dre-mir-17a-2, dre-mir-92a-2, tardbp, tardbpl, c13h9orf72	Knockout, insertion, chromosomal rearrangements	[81, 82, 203–206]
	Frog (Xenopus tropicalis)	tyr, six3	Knockout	[207]
	Pig (Sus scrofa)	gdf8, p65	Knockout, insertion	[208]
	Mouse (Mus musculus)	tet1, tet2, tet3, sry, uty, rosa26, hprt, egfp, th, rheb, uhrf2	Knockout, insertion	[83, 144, 209, 210]
	Rat (Rattus norvegicus)	dnmt1, dnmt3a, dnmt3b, tet1, tet2, tet3, mc3r, mc4r	Knockout, insertion	[144, 145, 211]
	Arabidopsis (Arabidopsis thaliana)	pds33, fls2, bri1, jaz1, gaj, chl, chl2, 5g13930	Knockout, insertion	[87, 88, 149]
	Tabacco (Nicotiana benthamiana)	Pds	Knockout, insertion	[88, 89]
	Rice (Oryza sativa)	ods, badh2, mrk2, 02g23823, roc5, spp, ysa, myb1, cao1, lazy1, sweet11, sweet14	Knockout, insertion	[86, 150, 152]
	Wheat (Triticum aestivum)	Mlo	Knockout	[86]

Laboratory animals, such as mouse and rat, are considered the most important model objects for the investigation of human diseases, basic research of the structure and function of genes and regulation of their expression, as well as in pharmacology and toxicology. Previously, mouse lines with knockout of specific genes were produced by homologous recombination in embryonic stem cells [1, 83], as well as by insertional mutagenesis [135, 136]. These are very time- and labor- consuming experiments, and generation of double knockout animals is a more difficult task. The CRISPR- Cas9-based genome editing technology is a faster and less labor-intensive way to do the job in a single step. Targeted injection of site specific nucleases into a single cell zygote causes double-strand DNA breaks at the target locus [137-139]. These breaks are repaired via the nonhomologous end joining mechanism that leads to the generation of mutant rats and mice carrying deletions or insertions at the cleaved site [140, 141]. Upon addition of a donor plasmid or oligonucleotide, the breaks can be repaired through the high precision homologous recombination mechanism that enables the production of animals carrying target DNA inserts [83, 142, 143].

Genome editing using CRISPR/Cas9 makes possible the introduction of mutations both into one gene and into a few genes at once. It was demonstrated that CRISPR-Cas9 generates, with a high efficiency, mutations in five genes simultaneously in mouse embryonic stem cells, and injection of *cas9 *mRNA and sgRNAs targeting the *Tet1 *and *Tet2 *genes into a mouse zygote generates animals with biallelic mutations in both genes with an efficiency of 80% [83]. Similar results were obtained in experiments in rats, with both mice and rats stably inheriting identifiable mutations [144, 145]. Furthermore, effective correction of a mutation in the *Crygc *gene in mice with a dominant form of cataract induced by this mutation was performed [146]. Generation of model rodents carrying specific mutations in several loci makes it possible to analyze the functions of genes belonging to gene families with redundant functions as well as epistatic interactions of genes. Data combining information on knockout of a certain mouse gene are available on the IMPC site (International Mouse Phenotyping Consortium, https://www.mousephenotype.org/).

Genome editing using TALENs and the CRISPR/Cas9 system is extensively used in plants. Targeted editing of plant genomes may be used to solve problems of both fundamental (investigation of gene function) and applied science (production of plants with new properties such as resistance to pathogens and herbicides, changes in metabolism, productivity, etc.) [147]. In this case, the protoplast transformation or* in planta *expression with *Agrobacterium tumefacients* (agroinfiltration) is primarily used for the delivery of genetically engineered constructs [148]. Gene knockouts and precise modification have been produced in plants, such as arabidopsis, wheat, rice, and tobacco [86, 88, 89, 149-153].

Editing of plant genomes using the TALEN system has, to date, been carried out in four model objects [70, 154-157]. Rice resistant to the pathogen of *Xanthomonas oryzae pv *serves as an example of a plant that acquired new properties due to genome editing using the TALEN system. A double-strand break was introduced into the wild-type pathogen TAL effector recognition site at the locus of the *Os11N3 *gene using artificial TALENs. In this way, plants resistant to infection by *X. oryzae pv *were produced [155].

## ALTERNATIVE WAYS TO USE TALE AND CRISPR/CAS9

Deciphering the recognition code between TALE proteins and target nucleotide sequences, as well as developing methods to generate artificial DNA-binding domains based on this code, has allowed scientists to construct chimeric proteins capable of acting directly on the genome. These proteins are composed of DNA-binding and effector domains. Nuclease domains are mainly used as the effector domain; however, in a number of studies, chimeric proteins were generated that contained, besides the DNA-binding domain, recombinase, histone methyltransferase, and histone deacetylase domains and domains that activate or suppress gene expression. These chimeric proteins have enormous prospects for application both in applied and in fundamental science. The CRISPR/Cas9 system is modified similarly: a certain effector domain, e.g., a transcriptional activator or repressor, the GFP fluorescent protein, etc, is attached to the catalytically inactive Cas9 protein.

**Regulation of gene expression using the TALE and CRISPR/Cas9 systems**

For targeted activation of gene expression, constructs containing the TALE DNA-binding domain and the synthetic VP64 domain [158], TALE-TF, are used. Once in the nucleus, a chimeric protein binds to a target nucleotide sequence and the VP64 domain attracts endogenous activators of gene expression [159]. In this case, the target gene expression is statistically significantly increased, which is usually confirmed by real time PCR. Activation of noncoding genes is also possible, e.g., the genes of miRNAs [160]. Suppression of the target gene expression can be achieved using chimeric proteins containing the KRAB [161] or SRDX [162] domains.

A possible therapeutic application of TALE-TF is the targeted regulation of the expression of the genes associated with human diseases. To test this approach, a strategy was used to increase the expression level of the *FXN *gene that encodes the frataxin protein. Expansion of GAA trinucleotide repeats in this gene leads to the development of Friedreich’s ataxia. In this case, the protein structure does not change but its expression is reduced. It was demonstrated that the *FXN *gene expression in human fibroblasts could be increased using TALE-TF, despite an increased number of the trinucleotide repeats [163].

Activation of endogenous gene expression avoids the use of ectopic overexpression of the reprogramming factors Oct4, Sox2, Klf4, and c-Myc (OSKM) in producing induced pluripotent stem cells. As a result, induced pluripotent stem cells can be produced that do not contain transgenes and, respectively, the risk of insertional mutagenesis, which arises when using lentiviral vectors expressing OSKM, can be reduced. For example, reprogramming of mouse embryonic fibroblasts to a pluripotent state was achieved through targeted activation of the expression of the *Oct4 *and *Nanog *genes under the influence of TALE-TFs containing the VP64 domain [164].

More recently, transcription factors were generated for the targeted regulation of gene expression in response to an external chemical stimulus. These factors consist of the TALE DNA-binding domain and ligandbinding domain of the steroid hormone receptor. When a ligand (ecdysone) enters the cell, dimerization of the ligand-binding domain and, respectively, activation of the target gene expression occur [165].

A recently developed system of light-inducible transcriptional effectors (LITEs) is a combination of two, very promising trends in modern biotechnology: optogenetics and genomic engineering. This system consists of two parts. The first is the TALE DNA-binding domain connected to a light-sensitive domain, cryptochrome 2 (CRY2), isolated from *Arabidopsis thaliana*. The second is the VP64 transcriptional activator coupled with CIB1, which is able to interact with CRY2. CRY2 alters its conformation under the blue light irradiation and binds to CIB1, thereby attracting VP64 to the target site [166]. A study by Konermann *et al*. [166], who developed the LITE system, demonstrated a statistically significant increase in the expression of some genes both in mouse neurons *in vitro *and in the brain* in vivo*. They also proposed a system in which the VP64 domain is replaced by methyltransferase or deacetylase capable of modifying histones.

An interesting application of targeted transcriptional regulation by TALE-TF is the development of genetic logic circuits inside the cell based on the interaction of several TALE-TFs with each other’s promoters and with a reporter gene and the promoters of the factors that regulate expression. Based on this approach, logical NOT-OR [167] and AND [168] circuits were produced inside cells.

Catalytically inactive dCas9 or dCas9 coupled with factors regulating gene expression also allows one to activate or repress transcription in human, bacterial, and yeast cells [112-116]. For this purpose, the *E. coli* omega-subunit of RNA polymerase [113], tandem copies of the viral VP64 protein, and the KRAB domain can be used [112, 115]. For example, highly specific silencing of the *CD71 *and *CXCR4 *genes (at the level of 60–80%) as well as effective knockdown of the *TEF1* locus in yeast were achieved [112]. Furthermore, multiplex activation/repression of the promoters of several genes was achieved, with the regulation type (positive or negative) being controlled by the target position in the gene promoter [114, 115]. Therefore, the CRISPR/ Cas9 system can be used as a modular platform that binds a given nucleotide sequence and attracts protein factors to it, thereby opening up opportunities of using this system as the main method for a precise regulation of gene expression in eukaryotic cells.

**Imaging of internal genomic loci using the TALE and CRISPR/Cas9 systems**

Chromatin organization and dynamics are known to play a decisive role in the regulation of genome activity. However, it is extremely difficult to obtain images of functional genomic loci in living cells. The use of the TALE and CRISPR/Cas9 systems opens up new possibilities for solving this problem.

Target DNAs in dynamics were visualized using constructs containing the TALE DNA-binding domain and a fluorescent protein [169-171]. This approach allows one to study the spatial and temporal organization of repeated genomic elements, including centromeric and telomeric repeats.

A method for imaging repetitive elements in the telomeres and the coding genes in living cells was developed using the endonuclease-deficient Cas9 protein labeled with EGFP and structurally optimized sgRNAs [117]. The repetitive and nonrepetitive elements in the* MUC4 *and *MUC1 *genes responsible for the production of various forms of mucin, which is a component of the protective mucus in various epithelial tissues and important in malignancy, were visualized in RPE, HeLa, and UMUC3 tumor cell lines [117]. Therefore, a possibility emerges to monitor the number of gene copies in living cells. The dynamics of telomere elongation and degradation, subnuclear localization of the *MUC4* loci, and cohesion of the replicated *MUC4 *loci on sister chromatids and their changing behavior during mitosis were observed using this method [117]. This strategy has significant potential for the study of the conformation and dynamics of native chromosomes in living human cells.

**Chimeric recombinases and transposases as an alternative to TALEN**

Recombinases and transposases are an alternative to TALEN in genome editing. Their advantages include the lack of dependence on the intracellular repair mechanisms. These enzymes also perform cleavage and ligation at target sites, and respectively in this case, no accumulation of double-strand breaks, which may lead to cell death, occurs. In addition, recombinases and transposases insert donor DNA into the genome, which simplifies detection of their activity. The disadvantage of these chimeric enzymes is a fairly high level of off-target effects [172]. A catalytic domain of Gin recombinase [173, 174] or piggyBack transposase [175] is used as an effector domain. The TALE recombinase activity was demonstrated using a reporter gene, the promoter of which was specifically cut out by Gin recombinase. The possibility to edit the genome using transposase was demonstrated in the case of the *CCR5* locus.

## CONCLUSIONS

The development of the TALEN and CRISPR/Cas9 systems is an important step in the progress achieved in modern genomic engineering. The emergence of these systems, due to their low cost and simplicity, has become a powerful impetus to the development of both fundamental and applied science. Prospects for the use of these systems in a variety of areas ranging from the food industry to personalized medicine are really amazing. However, until now, some problems have remained unresolved that are related to specificity and safety (due to possible off-target effects), delivery methods in therapeutic applications, and there is no answer to the question as to which of these systems combines the highest efficiency and safety?

The use of the CRISPR/Cas9 system has a number of advantages over the ZFN and TALEN based methods: it is much easier to produce, it is more efficient, and is suitable for high-performance and multiplex genome editing in a variety of cell lines and in living organisms. To refocus it on a new target needs only replacing the 20-nucleotide guide sequence of sgRNA. Also, Cas9 causes a break strictly between the 17th and 18th nucleotides in the target sequence (counting from the 5’-end of the spacer), i.e. at a distance of three nucleotides from the PAM. Moreover, simultaneous editing of several genes is greatly simplified by introducing a combination of sgRNAs. The use of nickase and modification of the sgRN A construction for a more accurate target recognition in the genome allow researchers to avoid undesired off-target effects.

The TALEN system is more labor-consuming, it takes more time to construct compared to CRISPR/ Cas9. However, there are now methods of automated design of TALEN-expressing constructs, which allows their efficient production on a commercial scale. Also, the fact that TALENs cause breaks only upon dimerization of the FokI domain, i.e. in pairs, increases the specificity and reduces the risk of off-target effects.

To date, there is no definitive answer to the question of which of the systems should be used. A detailed comparison of the two systems, with each having its own features, is required. It is quite conceivable that a universal answer to this question will never be found, and for each particular case, it will be necessary to test different variants and to choose among them those that are most appropriate to the research goals.

## Abbreviations

TALENs: transcription activator-like effector nucleases
CRISPR: clustered regulatory interspaced short palindromic repeats/Cas9
PAM: protospacer adjacent motif
sgRNA: single guide RNA
crRNA: CRISPR RNA
tcacrRNA: trans-activating CRISPR RNA
SpCas9: Streptococcus pyogenes Cas9
pre-crRNA: poly-spacer precursor crRNA
